# Development and validation of a frailty index in the Longitudinal Aging Study Amsterdam

**DOI:** 10.1007/s40520-016-0689-0

**Published:** 2016-11-28

**Authors:** Emiel O. Hoogendijk, Olga Theou, Kenneth Rockwood, Bregje D. Onwuteaka-Philipsen, Dorly J. H. Deeg, Martijn Huisman

**Affiliations:** 1 Department of Epidemiology and Biostatistics, EMGO+ Institute for Health and Care Research, VU University Medical Center, Amsterdam, The Netherlands; 2 Division of Geriatric Medicine, Department of Medicine, Dalhousie University, Halifax, NS Canada; 3 Department of Public and Occupational Health, EMGO+ Institute for Health and Care Research, VU University Medical Center, Amsterdam, The Netherlands; 4 Department of Sociology, VU University, Amsterdam, The Netherlands

**Keywords:** Frail elderly, Deficit accumulation, Frailty index, Longitudinal study, Mortality

## Abstract

**Background:**

Frailty is a state of increased vulnerability to adverse outcomes. The frailty index (FI), defined by the deficit accumulation approach, is a sensitive instrument to measure levels of frailty, and therefore important for longitudinal studies of aging.

**Aims:**

To develop an FI in the Longitudinal Aging Study Amsterdam (LASA), and to examine the predictive validity of this FI for 19-year mortality.

**Methods:**

LASA is an ongoing study among Dutch older adults, based on a nationally representative sample. A 32-item FI (LASA–FI) was developed at the second LASA measurement wave (1995–1996) among 2218 people aged 57–88 years. An FI score between 0 and 1 was calculated for each individual. The LASA–FI included health deficits from the physical, mental and cognitive domain and can be constructed for most LASA measurement waves. Associations with 19-year mortality were assessed using Kaplan–Meier curves and Cox proportional hazards models.

**Results:**

The mean LASA–FI score was 0.19 (SD = 0.12), with a 99% upper limit of 0.53. Scores were higher in women than men (women = 0.20, SD = 0.13 vs. men = 0.17, SD = 0.11, *p* < 0.001). The average age-related increase in the log-transformed LASA–FI score was 3.5% per year. In a model adjusted for age and sex, the FI score was significantly associated with 19-year all-cause mortality (HR per 0.01 = 1.03, 95% CI 1.03–1.04, *p* < 0.001).

**Discussion/conclusions:**

The key characteristics of the LASA–FI were in line with findings from previous FI studies in population-based samples of older people. The LASA–FI score was associated with mortality and may serve as an internal and external reference value.

## Introduction

Frailty is a state of increased vulnerability to adverse outcomes, such as falls, functional decline, hospitalization and death [1]. As the clinical importance of the concept of frailty is increasingly recognized, it is of major importance to identify frail older adults [2]. Many operational definitions of frailty exist [3]. One of the most widely used is the deficit accumulation approach, also known as the frailty index (FI). It involves the accumulation of diseases, symptoms, signs, disabilities or any deficiency in health with age, based on the idea that a greater number of health deficits indicate higher frailty [4]. Although health deficits increase with age, the FI characterizes age-related decline in health more efficiently than does chronological age [5]. Moreover, the FI has been shown to be a better predictor of adverse outcomes than chronological age [6] and even some other indices of biological age [7].

The items included in the FI are not fixed. As long as certain requirements are met, such as the type and number of health deficits included (it is recommended to include at least 30 health deficits representing several organ systems), it does not matter what combinations of health deficits are used [8]. This flexibility allows an FI to be constructed retrospectively in almost any dataset that includes comprehensive information on health and functioning.

The application of an FI may have added value for longitudinal studies in older populations. For these studies, a valid and sensitive frailty instrument is important so that the impact of frailty on various outcomes can be studied, as can its trajectory. In addition, it is imperative to apply the FI in different studies, to be able to compare its characteristics across different countries and settings. Until now, very few studies on the FI made use of data over an extended time period, and most studies were performed in North America [9–11].

The Longitudinal Aging Study Amsterdam (LASA) [12, 13], an ongoing study among Dutch older adults, is among the few European studies with a very long follow-up time. So far, the FI has not yet been constructed with LASA data. Therefore, the aim of this study was to develop and validate an FI in LASA. We described its characteristics and studied its relationship with chronological age. Since the validity of any frailty instrument largely depends on its ability to predict adverse outcomes, and in particular death, we validated this FI for 19-year all-cause mortality.

## Methods

### Design and study sample

LASA is an ongoing study on physical, emotional, cognitive and social functioning of older adults in the Netherlands. Details on the sampling and data collection of LASA have been published elsewhere [12, 13]. In summary, a nationally representative survey was conducted in 1992–1993 among 3107 respondents between the ages of 55 and 85. Follow-up measurements are collected approximately every 3 years. Data are collected in a face-to-face main interview in the respondent’s home by trained interviewers. During the main interview, respondents are asked to participate in a subsequent medical interview. After consent, a separate visit is scheduled in which clinical measurements are administered and additional questions are asked. The study received approval by the medical ethics committee of the VU University medical center. Signed informed consent was obtained from all study participants.

For the current study, data were used from the main interview of the second LASA measurement wave (1995–1996). Since various instruments were changed or included after the first LASA measurement wave, the second wave was more suitable to use than the first wave. Of the 2302 participants in the main interview, 84 (3.6%) were excluded because frailty level could not be identified due to missing data. This resulted in a final sample of 2218 persons aged 57–88 years.

### Frailty index construction

For the construction of the FI (LASA–FI) we followed the standard procedure described by Searle et al. [8]. Health deficits were included in the LASA–FI, if they (a) were biologically meaningful in representing several organ systems, and (b) were accumulating with age, and not becoming too prevalent at some younger age, and (c) did not contain too many missing values at item level (<5%), and (d) were available in the main interview of LASA at different measurement waves (to have the opportunity to study changes in LASA–FI score in future research).

We screened all questionnaires from the LASA main interview. From 34 potential variables we excluded two variables (hearing and vision), because they were not included in the LASA main interview at all subsequent measurement waves. Thus, 32 health deficits from the physical, mental and cognitive domain were used to construct the LASA–FI. Variables included self-reported chronic conditions: cardiac disease, peripheral arterial disease, stroke, diabetes, lung disease, cancer, arthritis, hypertension, a maximum of two other diseases and incontinence [14]; functional limitations: the ability to walk 15 stairs without resting, to (un)dress self, to sit and stand up from a chair, to cut own toenails, to walk outside for 5 min without stopping and to use public transportation [15]; self-rated health: the questions “How is your health in general?” and “How is your health compared to other people of your age?” [16]; six items from the CES-D depression scale: the extent to which people feel depressed, feel everything is an effort, feel happy, feel lonely, enjoy life and could not get going [17]; physical activity: based on the LASA physical activity questionnaire (LAPAQ) [18]; self-reported memory complaints [19]; four items from the Mini-Mental State Examination (MMSE): orientation in time, orientation in place, attention and recall [20]; and physical performance measured by gait speed [21]. See Table 1 for an overview of all included variables and cutoff values. All deficits were scored between 0 and 1, where 0 indicates the absence of the deficit and 1 the presence of a deficit.

**Table 1:** Overview of the variables included in the frailty index

No.	Deficit	Cutoff values
1	Cardiac disease	No = 0, yes = 1
2	Peripheral arterial disease	No = 0, yes = 1
3	Stroke	No = 0, yes = 1
4	Diabetes	No = 0, yes = 1
5	Lung disease	No = 0, yes = 1
6	Cancer	No = 0, yes = 1
7	Arthritis	No = 0, yes = 1
8	Hypertension	No = 0, yes = 1
9	Other chronic disease 1	No = 0, yes = 1
10	Other chronic disease 2	No = 0, yes = 1
11	Incontinence	No = 0, yes = 1
12	Walk up/down staircase 15 steps without resting	Yes = 0, yes, with some difficulty = 0.25, yes, with much difficulty = 0.50, only with help = 0.75, No = 1
13	Dress/undress self	Yes = 0, yes, with some difficulty = 0.25, yes, with much difficulty = 0.50, only with help = 0.75, no = 1
14	Sit down/stand up from chair	Yes = 0, yes, with some difficulty = 0.25, yes, with much difficulty = 0.50, only with help = 0.75, no = 1
15	Cut own toenails	Yes = 0, yes, with some difficulty = 0.25, yes, with much difficulty = 0.50, only with help = 0.75, no = 1
16	Walk outside 5 min without stopping	Yes = 0, yes, with some difficulty = 0.25, yes, with much difficulty = 0.50, only with help = 0.75, no = 1
17	Use of transportation	Yes = 0, yes, with some difficulty = 0.25, yes, with much difficulty = 0.50, only with help = 0.75, no = 1
18	How is your health in general?	Excellent = 0, good = 0.25, fair = 0.50, sometimes good/bad = 0.75, Poor = 1
19	How is your health compared to other people of your age?	Much better/a little better = 0, just as good = 0.33, a little worse = 0.66, much worse = 1
20	Feel depressed (CES-D)	Rarely or never = 0, some of the time = 0.33, occasionally = 0.66, mostly or always = 1
21	Feel everything is an effort (CES-D)	Rarely or never = 0, some of the time = 0.33, occasionally = 0.66, mostly or always = 1
22	Feel happy (CES-D)	Mostly or always = 0, occasionally = 0.33, some of the time = 0.66, rarely or never = 1
23	Feel lonely (CES-D)	Rarely or never = 0, some of the time = 0.33, occasionally = 0.66, mostly or always = 1
24	Enjoy life (CES-D)	Mostly or always = 0, occasionally = 0.33, some of the time = 0.66, rarely or never = 1
25	Could not get going (CES-D)	Rarely or never = 0, some of the time = 0.33, occasionally = 0.66, mostly or always = 1
26	Physical activity (LAPAQ)	High (five or more activities) = 0, medium (3–4 activities) = 0.33, low (1–2 activities) = 0.66, no activities = 1
27	Memory complaints	No = 0, yes = 1
28	Orientation time (MMSE)	Five correct = 0, one wrong = 0.50, two or more wrong = 1
29	Orientation place (MMSE)	Five correct = 0, one wrong = 0.50, two or more wrong = 1
30	Attention (MMSE)	Five correct = 0, one or two wrong = 0.50, three or more wrong = 1
31	Recall (MMSE)	Three correct = 0, two correct = 0.50, one or zero correct = 1
32	Gait speed (6 m)	Normal = 0, slow (>10 s) or physical unable = 1

We did not calculate a frailty score for participants with more than 20% missing variables of the LASA–FI. This commonly used criterion allows for maximum use of available data without excessive reliance on imputation procedures [22]. A frailty score was calculated for each participant by dividing the sum of the health deficit scores by the total number of health deficits measured. This resulted in a score between 0 (no deficits present) and 1 (all deficits present). For example, if a person has six points out of 32, the LASA–FI score was 6/32 = 0.19. The LASA–FI may be used as a continuous score, or as a dichotomous variable by applying a generally used cutoff point of ≥0.25 to indicate frailty [23]. The SPSS syntax for the construction of the LASA–FI will be provided at the LASA study website (www.lasa-vu.nl).

### Mortality

Mortality status was retrieved from registers of the municipalities where respondents were living. All deaths that occurred between the baseline measurement and July 1, 2015, were recorded (99.7% ascertainment for the current sample).

### Statistical analysis

The distribution of the LASA–FI was assessed using a histogram. Descriptive statistics, including the mean FI score and standard deviations, were calculated for the total study population and by sex. Differences between men and women were determined using *t* test statistics. LASA–FI scores in relation to age were studied in several ways. First, mean FI scores and frailty prevalence were reported by 5-year age groups. Second, mean FI scores were plotted versus age, stratified by sex. Finally, a linear regression analysis with age as independent variable and the natural log of the LASA–FI as dependent variable was performed to estimate the increase in the LASA–FI score with age.

Kaplan–Meier curves were constructed to estimate 19-year survival for categories of the LASA–FI score. Bivariate and multivariable Cox proportional hazard models were fitted to study the association between the LASA–FI score and 19-year all-cause mortality. Hazard ratios (HR) and 95% confidence intervals (95% CI) were reported for the total population, by sex and by age group (<80 years vs. ≥80 years). Multivariable models were adjusted for age and sex (if applicable). Survivors were censored at the end of the follow-up (July 1, 2015). All analyses were performed in SPSS 22 (IBM Corp, Armonk, NY, USA).

## Results

**Fig. 1:**
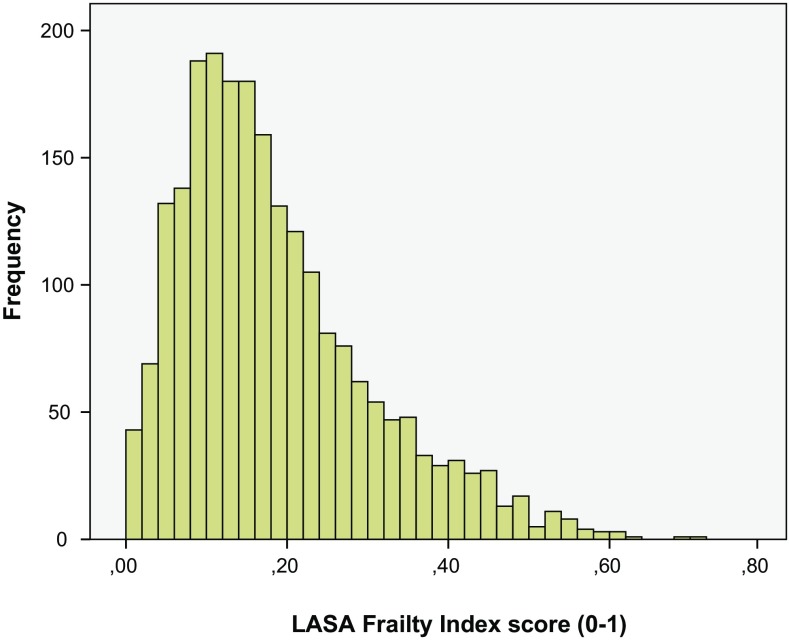
Distribution of the frailty index at baseline (*N* = 2218)

We were able to calculate the LASA–FI for 2218 people out of 2302 available respondents (96.4%). Of the 2218 people in the analytic sample, 2092 (94.3%) had no missing values on the 32 items of the LASA–FI, 103 had only 1 missing item (4.6%), and 23 had 2 to 6 missing items (0.1%). Figure 1 shows the distribution of the LASA–FI, which was skewed to the right.

Table 2 shows the LASA–FI scores for the total study population and stratified by age group and sex. The mean FI score was 0.19 (SD = 0.12), with a median of 0.16 and a range from 0.00 to 0.71. The 99% upper limit was 0.53. Mean FI scores were higher in women than in men (women = 0.20, SD = 0.13 vs. men = 0.17, SD = 0.11, *p* < 0.001). Table 2 also shows the frailty prevalence when applying the cutoff of 0.25 and higher. Overall, 23.9% of the respondents was considered to be frail. Frailty prevalence was higher in women (28.8%) than in men (18.4%).

**Table 2:** Frailty index score and frailty prevalence by age group

Age group	Frailty index score			Frailty prevalence (score ≥0.25)		
	Overall	Women	Men	Overall	Women	Men
	Mean (SD)	Mean (SD)	Mean (SD)	%	%	%
57–61 (*N* = 300)	0.13 (0.08)	0.13 (0.08)	0.12 (0.08)	8.3	7.7	9.0
62–66 (*N* = 432)	0.14 (0.09)	0.16 (0.10)	0.13 (0.07)	11.1	14.1	7.6
67–71 (*N* = 400)	0.15 (0.10)	0.17 (0.11)	0.13 (0.08)	13.5	18.1	8.2
72–76 (*N* = 342)	0.19 (0.11)	0.20 (0.120	0.17 (0.10)	22.2	26.2	17.6
77–81 (*N* = 354)	0.23 (0.13)	0.26 (0.13)	0.21 (0.11)	37.0	44.6	28.8
≥82 (*N* = 390)	0.27 (0.12)	0.30 (0.12)	0.24 (0.12)	50.0	61.5	37.9
Total (*N* = 2218)	0.19 (0.12)	0.20 (0.13)	0.17 (0.11)	23.9	28.8	18.4

**Fig. 2:**
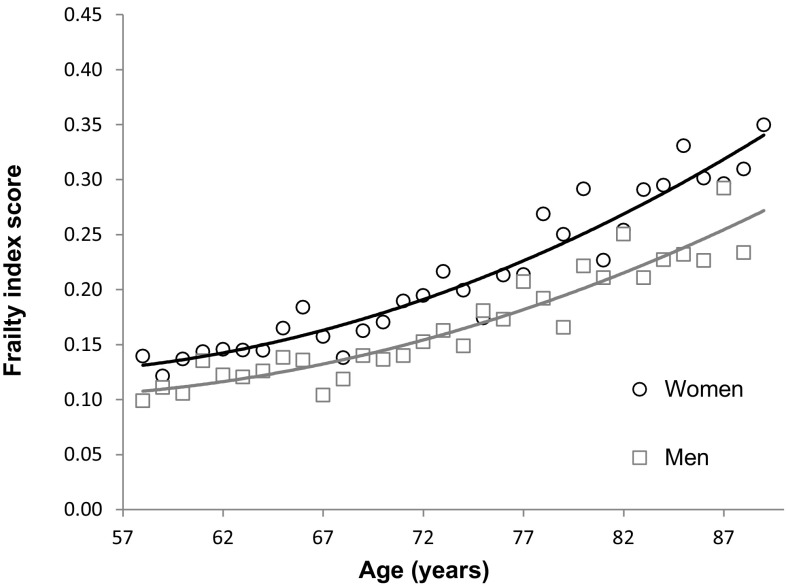
Average frailty index score by sex and age

Mean FI scores and frailty prevalence increased with age in both men and women (Fig. 2). Using the natural log of the FI in linear regression, the overall slope of the deficit accumulation in relation to age was 0.035 (SE = 0.002, *p* < 0.001), which means that the log-transformed FI score increased on average 3.5% per year.

**Fig. 3:**
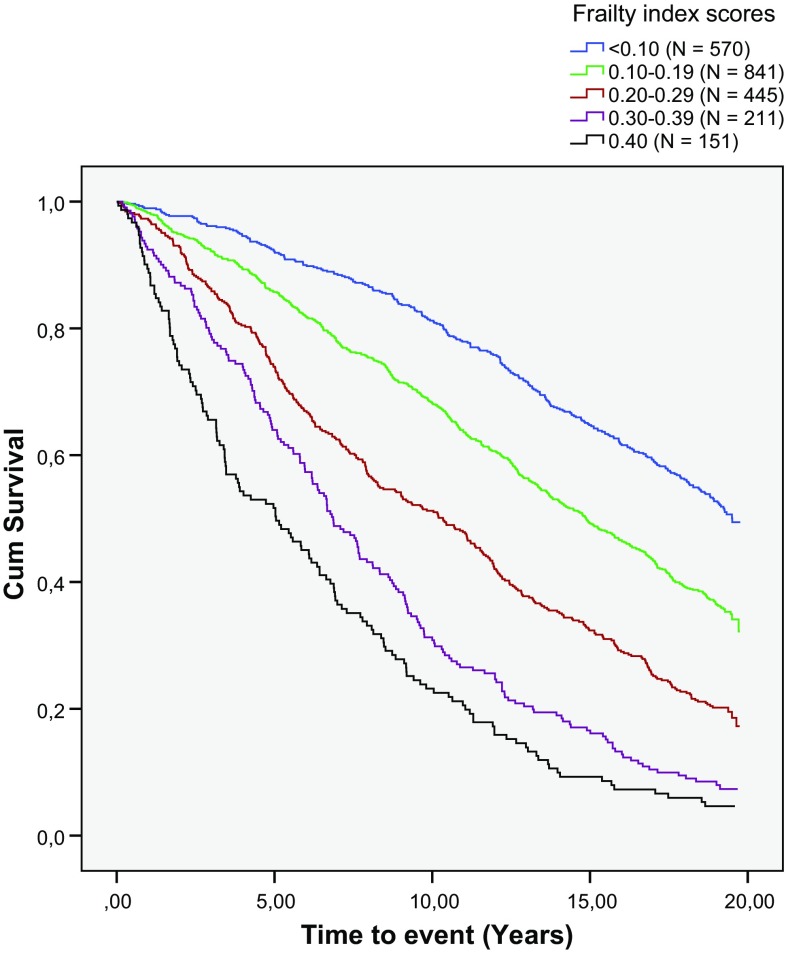
Kaplan–Meier curves according to frailty index score: proportions of people who survived plotted against time

Of the study sample of 2218 respondents, 1520 (68.5%) died during the 19-year follow-up. The median survival time was 13.1 years. People with higher LASA–FI scores had a lower probability of survival (Fig. 3). Table 3 shows the HRs for 19-year all-cause mortality (bivariate and multivariable analyses for the total sample and stratified by age group). In the bivariate Cox regression analyses, age, sex and the LASA–FI were each significantly associated with mortality. Each 0.01 increment in FI score remained associated with mortality after adjustment for age and sex (HR = 1.03. 95% CI 1.03–1.04). The association between the FI score and mortality seemed to be slightly stronger among men (HR = 1.04, 95% CI 1.03–1.05) than among women (HR = 1.02, 95% CI 1.02–1.03). The analyses stratified by age group (<80 years and ≥80 years) showed similar results.

**Table 3:** Cox regression analyses for the total sample and stratified by age group: hazard ratios for 19-year all-cause mortality

Variable	Unadjusted (bivariate)		Adjusted					
	Overall		Overall		Men		Women	
	HR	95% CI	HR	95% CI	HR	95% CI	HR	95% CI
Total sample (*N* = 2218)								
Age	1.12	1.11–1.13	1.11	1.10–1.12	1.10	1.09–1.11	1.12	1.11–1.13
Sex (male)	1.43	1.30–1.59	1.89	1.70–2.01	–	–	–	–
Frailty index^a^	1.04	1.04–1.05	1.03	1.03–1.04	1.04	1.03–1.05	1.02	1.02–1.03
Age < 80 (*N* = 1685)								
Age	1.13	1.12–1.14	1.12	1.11–1.14	1.11	1.09–1.13	1.14	1.12–1.16
Sex (male)	1.49	1.31–1.69	1.90	1.67–2.16	–	–	–	–
Frailty index^a^	1.03	1.03–1.04	1.03	1.02–1.04	1.04	1.03–1.05	1.03	1.02–1.03
Age ≥ 80 (*N* = 533)								
Age	1.10	1.06–1.15	1.09	1.05–1.14	1.09	1.03–1.15	1.11	1.05–1.18
Sex (male)	1.39	1.17–1.65	1.90	1.58–2.30	–	–	–	–
Frailty index^a^	1.03	1.02–1.03	1.03	1.03–1.04	1.04	1.03–1.06	1.02	1.01–1.04

^a^The frailty index hazard ratios measure a 0.01 change on the index

## Discussion

In this study, we successfully constructed an FI in LASA. We described its characteristics and validated the LASA–FI for 19-year all-cause mortality. The key characteristics of the LASA–FI are consistent with findings from previous studies in population-based samples of older adults: The distribution of the LASA–FI is skewed to the right, the LASA–FI score increases with chronological age, and the average LASA–FI scores are higher in women than in men [6, 8]. Also, the 99% upper limit was below 0.7, as in previous studies [8, 24]. In the current study, the average rate of deficit accumulation with age was 0.035, which was comparable to other longitudinal studies in community-based samples (mean rate of 0.029) [25].

This was one of the first European studies in which the FI was validated for mortality over an extended time period. Our results showed that the LASA–FI was associated with 19-year all-cause mortality. The Kaplan–Meier curves clearly demonstrated that the risk for mortality increased with a higher LASA–FI score. For example, among people with a LASA–FI score <0.10 only 19% died within 10 years, whereas 77% of the people with a LASA–FI score ≥0.40 died within 10 years.

LASA is based on a nationally representative sample of older adults in the Netherlands. Therefore, the LASA–FI may serve as a reference value for other Dutch studies in more specific populations. In addition, the LASA–FI may provide opportunities for comparisons with patient groups in healthcare settings, as the variables included in this FI are often part of routine data collection (e.g., diseases, functional limitations, MMSE). Furthermore, the LASA–FI may be used to select internal reference groups. For example, if researchers would like to select a healthy control group, people with the lowest LASA–FI scores may be selected, as these are the most healthy and stable older adults in the sample.

The LASA–FI also provides many opportunities for future research. First, it may be used as a predictor of adverse outcomes. In this study, we investigated the association with mortality. Further research on the predictive ability of the LASA–FI may be focused on other outcomes such as falls and healthcare use. The LASA–FI may also be studied in relation to the social domain, such as social support and social network characteristics [26]. Second, the LASA–FI may be used to study longitudinal changes in frailty. Previous frailty studies in LASA included another widely used frailty definition: the frailty phenotype [27–29]. This instrument defines frailty based on the number of the following criteria present: weight loss, weak grip strength, exhaustion, slow gait and low physical activity [30]. The frailty phenotype showed good predictive ability for functional decline and mortality [28]. However, as an outcome measure the frailty phenotype is less useful, because with only five items it lacks sensitivity to study changes in frailty states over an extended time period. This is in contrast with the FI, which has the potential to monitor changes in frailty over time in longitudinal studies [31]. Future research may explore the responsiveness of the LASA–FI and compare the predictive ability of the LASA–FI with other frailty definitions.

The current FI contains 32 health deficits from the physical, mental and cognitive domain. However, there is some flexibility with regard to the number and type of variables to be included in the LASA–FI. For some research questions, it may be necessary to exclude items. For example, if someone would like to study frailty in relation to cognitive outcomes, the cognitive items of the LASA–FI may be replaced by variables from other domains. Variables from the LASA medical interview may be considered for that purpose, such as peak flow, pain, body mass index and grip strength. However, it should be noted that this may reduce the sample size, as about 85% of the participants in the main interview agree to participate in this additional interview.

Since FIs can be derived from routinely available healthcare data (e.g., electronic medical records), the FI has great potential for use in clinical practice. Various studies have shown that the FI can be used to identify frail older patients who may benefit from healthcare interventions [32–34]. For example, this has recently been demonstrated in a study using routine data of over 900,000 primary care patient in the UK [32].

In conclusion, the key characteristics of the LASA–FI were in line with previously published FIs. The LASA–FI was significantly associated with mortality and may serve as an internal and external reference value. The instrument provides ample opportunities for future research, where the LASA–FI may be used as a predictor or outcome measure.
